# Virtual Screening for Biomimetic Anti-Cancer Peptides from *Cordyceps militaris* Putative Pepsinized Peptidome and Validation on Colon Cancer Cell Line

**DOI:** 10.3390/molecules26195767

**Published:** 2021-09-23

**Authors:** Jarinyagon Chantawannakul, Paninnuch Chatpattanasiri, Vichugorn Wattayagorn, Mesayamas Kongsema, Tipanart Noikaew, Pramote Chumnanpuen

**Affiliations:** 1 Mahidol Wittayanusorn School, 364 Salaya, Phuttamonthon District, Nakhon Prathom 73170, Thailand; kiwi.jarinyagon@gmail.com (J.C.); paninnuch.1@gmail.com (P.C.); 2 Department of Zoology, Faculty of Science, Kasetsart University, Bangkok 10900, Thailand; vichugorn.wa@ku.th (V.W.); fscimmk@ku.ac.th (M.K.); 3 Department of Biology and Health Science, Mahidol Wittayanusorn School, 364 Salaya, Phuttamonthon District, Nakhon Prathom 73170, Thailand; 4 Omics Center for Agriculture, Bioresources, Food and Health, Kasetsart University (OmiKU), Bangkok 10900, Thailand

**Keywords:** *Cordyceps militaris*, bioinformatics, colorectal cancer, chemotherapy, apoptosis

## Abstract

Colorectal cancer is one of the leading causes of cancer-related death in Thailand and many other countries. The standard practice for curing this cancer is surgery with an adjuvant chemotherapy treatment. However, the unfavorable side effects of chemotherapeutic drugs are undeniable. Recently, protein hydrolysates and anticancer peptides have become popular alternative options for colon cancer treatment. Therefore, we aimed to screen and select the anticancer peptide candidates from the in silico pepsin hydrolysate of a *Cordyceps militaris* (CM) proteome using machine-learning-based prediction servers for anticancer prediction, i.e., AntiCP, iACP, and MLACP. The selected CM-anticancer peptide candidates could be an alternative treatment or co-treatment agent for colorectal cancer, reducing the use of chemotherapeutic drugs. To ensure the anticancer properties, an in vitro assay was performed with “CM-biomimetic peptides” on the non-metastatic colon cancer cell line (HT-29). According to the 3-(4,5-dimethylthiazol-2-yl)-2,5-diphenyltetrazolium bromide (MTT) assay results from peptide candidate treatments at 0–400 µM, the IC_50_ doses of the CM-biomimetic peptide with no toxic and cancer-cell-penetrating ability, original *C. militaris* biomimetic peptide (C-ori), against the HT-29 cell line were 114.9 µM at 72 hours. The effects of C-ori compared to the doxorubicin, a conventional chemotherapeutic drug for colon cancer treatment, and the combination effects of both the CM-anticancer peptide and doxorubicin were observed. The results showed that C-ori increased the overall efficiency in the combination treatment with doxorubicin. According to the acridine orange/propidium iodine (AO/PI) staining assay, C-ori can induce apoptosis in HT-29 cells significantly, confirmed by chromatin condensation, membrane blebbing, apoptotic bodies, and late apoptosis which were observed under a fluorescence microscope.

## 1. Introduction

Colorectal cancer is the third most common cancer globally [1]. Even though the standard treatments, such as surgery and radiotherapy, can effectively treat several cancer cases, chemotherapy is the most common treatment performed on advanced metastasis diseases. [2]. However, chemotherapeutic drugs typically aim to destroy rapidly dividing cells and inadvertently attack healthy cells and tissues, which results in a considerable number of unfavorable side effects [3]. The advent of modern molecular biology brought short peptides which could inhibit a wide range of microbes (bacteria and fungi) [4]. After a considerable number of peptides that expressed antimicrobial and antifungal activity were accumulated, cationic peptides with a low molecular weight were also shown to have special functions as anticancer peptides (ACPs). ACPs are a series of short peptides composed of 10–60 amino acids that can suppress tumor cell proliferation and expression [5]. ACPs have advantages over the conventional chemotherapy as they possess unique mechanisms, which lead to better inhibition of cell proliferation, migration, and angiogenesis [6]. ACPs have been reported to have a high cell penetration and low drug resistance; as such, the clinical application of ACPs is promising. Furthermore, the synthesis and modification of ACPs are inexpensive [7]. As a result, ACPs are becoming an alternative treatment or co-treatment with conventional chemotherapeutic agents in clinical use [8,9]

*Cordyceps militaris* (CM) has been widely used as a nutrient supplement in eastern Asia, and is extensively applied in modern medical therapeutic methods [10]. CM peptides are reported to have anticancer activities toward breast, lung, and bladder cancers, although the anticancer activities of CM peptides against colorectal cancer remains unclear [11]. Because of the time, laboring, and the exorbitant procedures that have to be conducted in order to identify CM ACPs, the computational method is an effective alternative to reduce expenses.

In this study, we proposed the use of support vector machine (SVM)-based predictors and the ensemble approach to identify the potential ACP candidates from the *Cordyceps militaris* peptide dataset. AntiCP utilized the amino acid composition and binary features, with SVM algorithm support, to classify the ACPs. The database of AntiCP was based on a dataset of 225 antimicrobial peptides (AMPs) with anticancer properties and random peptides from SwissProt (https://www.expasy.org/resources/uniprotkb-swiss-prot, accessed on 12 May 2021) [12]. Another SVM-based predictor, iACP, classified the ACPs based on sequence information (i.e., amino acid composition, dipeptide composition, atomic composition, and physiochemical properties) [13]. MLACP developed the use of a random forest and an SVM machine learning approach to predict ACPs using sequence information [14]. These predictors could effectively screen the potential ACPs from a considerable number of natural peptides.

Even though ACPs have many advantages, they also contain many shortcomings which diminish the peptides’ efficiency [15]. The reconstruction and modification of ACPs are needed to reduce their side effects and improve their therapeutical properties. The reconstructions are mainly main chain reconstructions and side chain modifications. The main chain reconstructions involve the change of natural and non-natural amino acids, whereas the side change modifications consist of cholesterol modification, phosphorylation, glycosylation, and palmitoylation [16,17]. The replacement of natural amino acids was utilized in this study as it had great significance on the structure and function of the peptides. The amino acid substitution also allowed us to examine the effects of each amino acid and dipeptide on the properties of the peptides [18].

To evaluate the anticancer activities of the anticancer candidate peptides, in vitro methods were utilized to confirm viability inhibition against colon cancer cell lines. The fluorescence measurement was also used to investigate the activity of peptides against the cells and identify the modes of action. Thus, our bioinformatic analysis provided ACP identification from nearly twenty thousand peptides, and the in vitro result showed the effects of peptides on cell lines.

## 2. Results

### 2.1. Putative Anticancer Peptides Screening Using Computational Method

**Figure 1:**
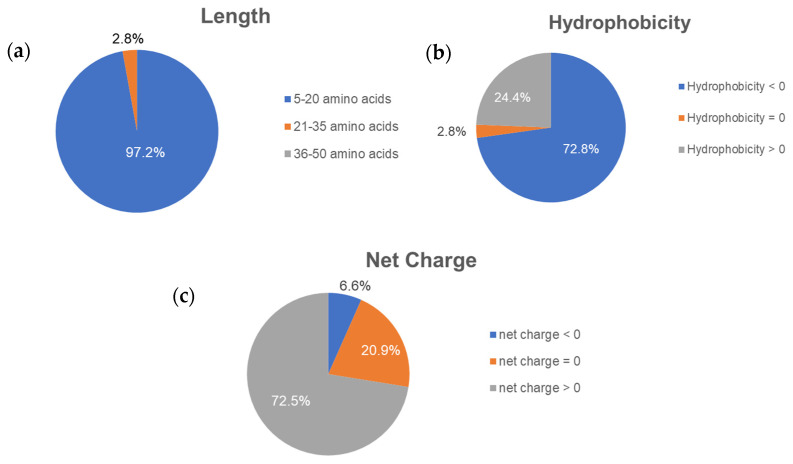
Percentage of the peptides’ properties regarding their (**a**) length, (**b**) hydrophobicity, and (**c**) net charge from 316 putative ACPs.

The peptides were predicted to be anti-cancer peptides (ACPs) if: the cut off criteria at the support vector machine (SVM) score was over 1.00 in AntiCP; the probability was over 0.5 in iACP; and the scores of both random forest (RF) and SVM probabilities were over 0.5. As a result, AntiCP predicted 20,525 ACPs; iACP predicted 3,739 ACPs; MLACP predicted 1,181 ACPs; and three prediction servers predicted 316 ACPs in common from 21,148 unique CM peptides. In terms of the peptides’ properties (Figure 1), the majority of putative ACPs were 5–20 amino acids long (97.2%), hydrophilic (72.8%), and cationic (72.5%).

ToxinPred (https://webs.iiitd.edu.in/raghava/toxinpred/protein.php, accessed on 12 May 2021) and MLCPP (http://www.thegleelab.org/MLCPP, accessed on 12 May 2021) were used to predict the modes of action of the putative ACPs. ToxinPred predicted the toxicity of peptides against cancerous cells, while MLCPP predicted the cell penetration ability of peptides against cancer. Both ToxinPred and MLCPP predicted 14 peptides to be toxic ACPs; 242 peptides to be cell-penetrating ACPs; 5 peptides to have both modes of action; and 55 peptides to have no mode of action. We were interested to investigate the difference in the effects between peptides with no modes of action and with both modes of action.

From all five peptides having both modes of action, TTMICLTCAR was among the predicted peptides with highest prediction scores (SVM score, RF obability, (RF-ACP), and SVM-ACP). As a result, TTMICLTCAR was the candidate for the original toxic and cancer cell-penetrating peptide named “CTP-ori”. CTP-ori had ten amino acids consisting of three polar uncharged threonines, two cysteines, and one positive charged arginine. CTP-ori was a hydrophilic, amphiphilic, and cationic peptide. To increase the prediction score, CTP-ori was re-designed by substituting certain amino acid residues using the peptide design option on AntiCP. CTP-ori was modified into a CM-biomimetic toxic and cancer cell penetration peptide, or “CTP-rds”, by replacing methionine and alanine with glycine and cysteine, respectively (TTGICLTCCR) (Table 1).

From all 55 peptides with no special mode of actions against cancer cells, VTFVLIAAK was the candidate for the original CM-biomimetic peptide named “C-ori”. C-ori had nine amino acids with double alanine residues, one polar uncharged threonine, one aromatic residue (phenylalanine), and a positive charged lysine (VTFVLIAAK). The physiochemical properties of C-ori were hydrophobic, amphipathic, and cationic. The modified CM-biomimetic peptide, C-rds, was the result of AntiCP’s modification by having the first valine and isoleucine replaced by phenylalanine and leucine, respectively (FTFVLLAAK) (Table 1).

In accordance with the bioinformatics prediction, we decided to synthesize four putative ACPs (TTMICLTCAR; TTGICLTCCR; VTFVLIAAK; and FTFVLLAAK).

**Figure 2:**
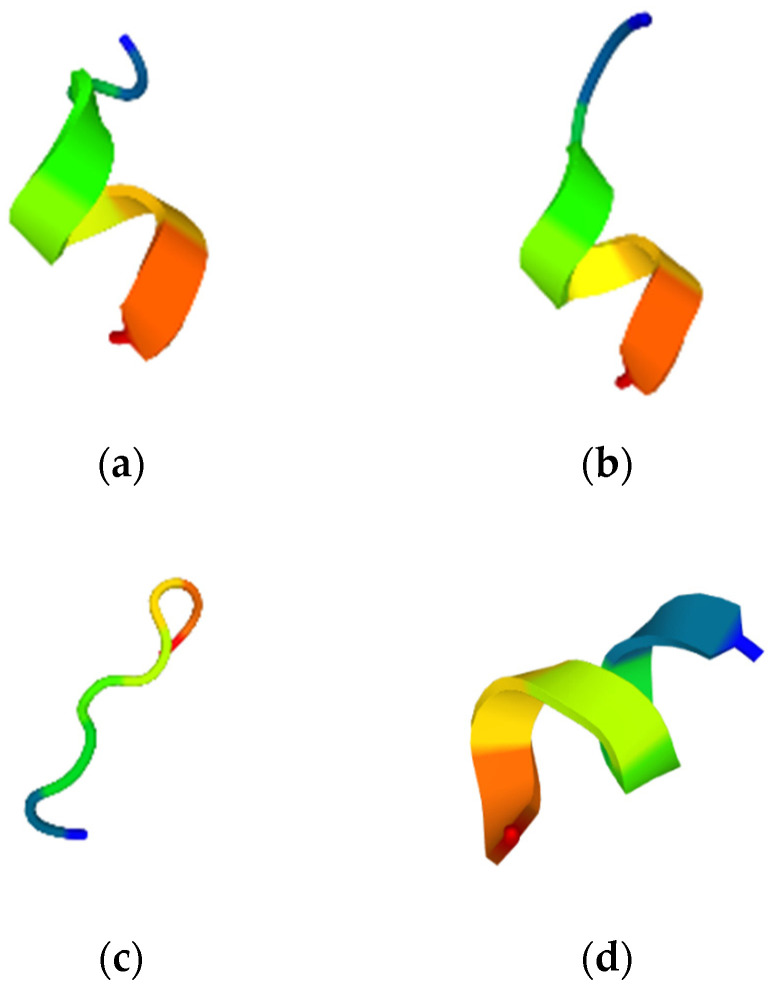
The molecular structure of: (**a**) the original CM-biomimetic toxic and cancer-cell-penetrating peptide, CTP-ori; (**b**) the modified CM-biomimetic toxic and cell-penetrating peptide, CTP-rds; (**c**) the original CM-biomimetic peptide, C-ori; (**d**) and the modified CM-biomimetic peptide, C-rds. The red end represents the C-terminal, and the blue end represents the N-terminal.

All selected peptides’ molecular structures were predicted using PEP-FOLD3.5. The C-ori was random coiled (Figure 2c), whereas the CTP-ori, CTP-rds, and C-rds were single helixes (Figure 2a,b,d).

### 2.2. The Inhibitory Effect of Selected Putative Anticancer Peptides against HT-29 Cell Line

**Figure 3:**
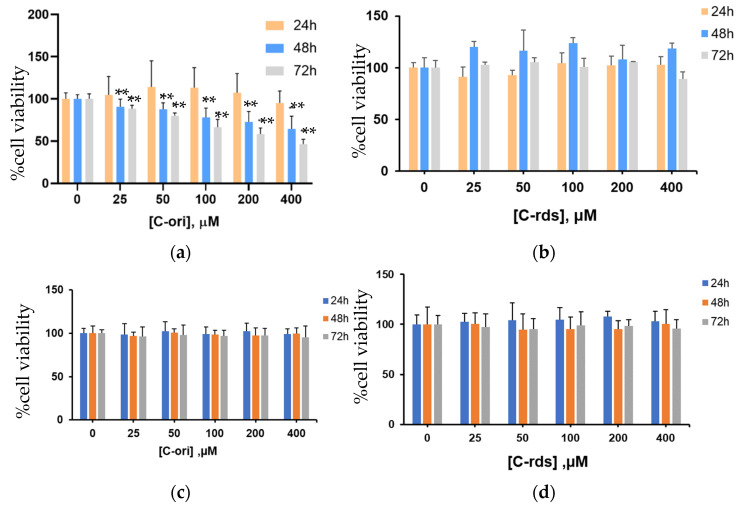
Inhibition of HT-29 cell viability treated by (**a**) C-ori and (**b**) C-rds. The cell viability of human dermal fibroblast cells treated by (**c**) C-ori and (**d**) C-rds. Cell viability was assessed by an MTT assay after a 24 h, 48 h, and 72 h incubation with drugs. The results shown are mean ± SD; *n* = 4 trials. (** p < 0.01).

The inhibitory effects of putative ACP candidates were investigated using an MTT cell viability assay. The results showed that C-ori decreased the cell viability of the HT-29 cell line in a dose-dependent manner with no effect on human dermal fibroblasts. The IC_50_ of C-ori was 114.9 ± 1.5 µM (Figure 3a). Contrarily, CTP-ori, CTP-rds (Supplementary Figure S1a,b), and C-rds (Figure 3b) showed no effect on the HT-29 cell line despite the increases in time and dose.

### 2.3. The Effects of the Original CM-Biomimetic Peptide and Doxorubicin against HT-29 Cell Line

**Figure 4:**
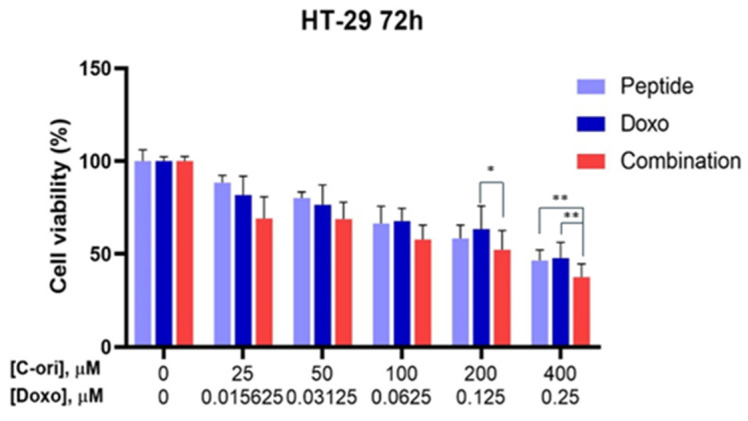
Combination effects of C-ori with doxorubicin on the HT-29 cell line. Cell viability was assessed using an MTT assay after a 72-hour incubation with drugs (* *p* < 0.05, ** *p* < 0.01).

The original CM-biomimetic peptide’s effects were compared with the conventional chemotherapeutic drug, doxorubicin, and the combination effects of both peptide and doxorubicin were observed. The IC_50_ of the doxorubicin ranged from 0.04 to 12 µM. According to Figure 4, the combination of the original CM-biomimetic peptide and doxorubicin showed a slightly increased effect against the HT-29 cell line.

### 2.4. The Effect of Original CM-Biomimetic Peptide and/or Doxorubicin on Apoptosis against HT-29 Cell Lines Using AO/PI Staining Assay

**Figure 5:**
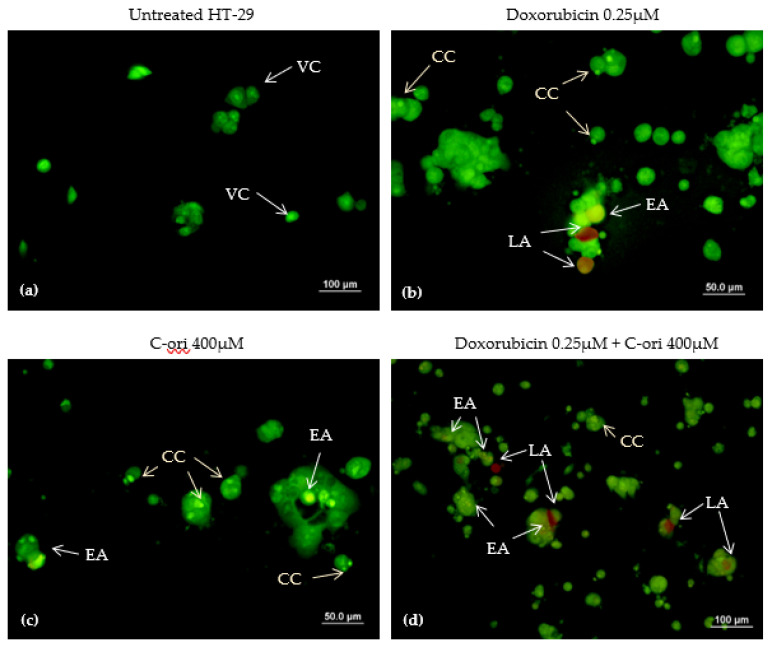
Acridine orange/propidium iodide double staining of HT-29 cells after 72 h exposure: (**a**) untreated (control); (**b**) 0.25µM doxorubicin; (**c**) 400µM C-ori; and (**d**) 0.25µM doxorubicin + 400µM C-ori. VC = viable cells; BL = membrane blebbing; EA = early apoptosis; LA = late apoptosis; CC = chromosome condensation. Magnification: 40× and 100×.

**Figure 6:**
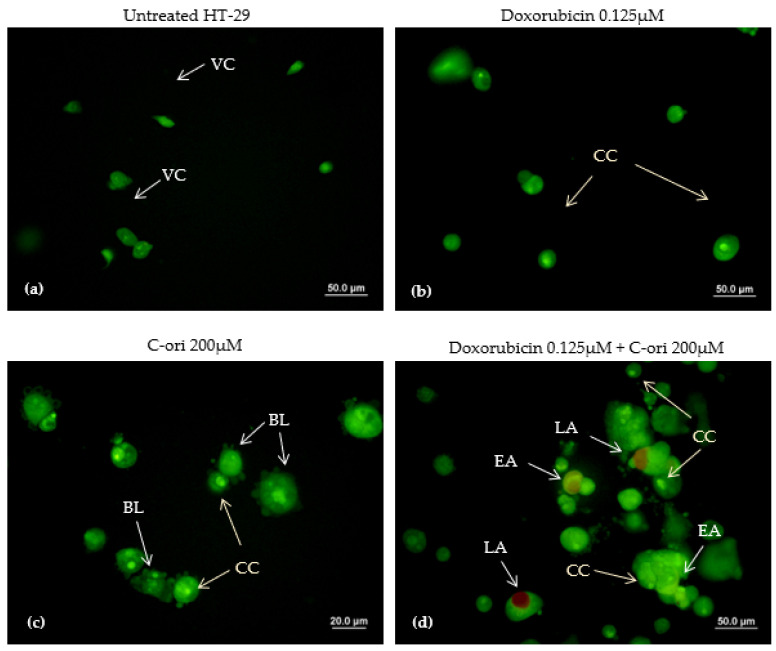
Acridine orange/propidium iodide double staining of HT-29 cells after 72 h exposure: (**a**) untreated (control); (**b**) 0.125µM doxorubicin; (**c**) 200µM C-ori; and (**d**) 0.125µM doxorubicin + 200µM C-ori. VC = viable cells; BL = membrane blebbing; EA = early apoptosis; LA = late apoptosis; CC = chromosome condensation. Magnification: 40× and 100×.

The apoptotic inductive effects of doxorubicin, C-ori, and the combination of both doxorubicin and C-ori on HT-29 were observed via morphological changes under a fluorescence microscope. The untreated HT-29 cells exhibited a rounded shape and an intact membrane displayed as a green fluorescence. Under the treated conditions, the morphology of HT-29 changed after the 72 h treatment of C-ori and 0.125 µM doxorubicin with the presence of cell membrane blebbing, early apoptosis, and chromatin condensation. The cells that underwent late apoptosis were observed as a reddish-orange fluorescence after 72 h of the combination of doxorubicin, C-ori, and 0.25 µM doxorubicin (Figure 5 and Figure 6).

## 3. Discussion

The ACPs acted against malignant cancer cells with either membrane-disrupting processes or mitochondrial disruptions [9,19]. The net charge, amphiphilicity, and hydrophobicity were the key physiochemical properties for the ACPs to successfully disrupt the negative-charged cancer membrane and penetrate the cells. All mentioned physiochemical properties are indicated by their amino acid compositions [20,21]. Each of the selected putative ACPs possessed distinct properties: CTP-ori (TTMICLTCAR) was a hydrophilic, amphiphilic, and cationic peptide which was predicted to disturb the cancer cells with cytotoxicity and membrane-penetrating abilities; CTP-rds (TTGICLTCCR) shared the same characteristics with CTP-ori; C-ori (VTFVLIAAK) was a hydrophobic, amphiphilic, and cationic random coiled peptide. This peptide showed no toxicity nor cell-penetration abilities against the cancer cell, and was predicted by prediction servers named ToxinPred and MLCPP, respectively; C-rds (FTFVLLAAK) was a hydrophobic, amphiphilic, and cationic single helix peptide, and it possessed a potential non-toxic ability to penetrate the cancer cells.

Replacing the valine in C-ori with phenylalanine may have enhanced the cell membrane targeting, as phenylalanine is abundant in primary tumor membranes, and leucine residues are highly present in other reported ACPs [20,22,23,24,25]. Because of these reasons, C-rds was predicted with a higher prediction score in the in silico method. Nevertheless, the in vitro method indicated that only C-ori was effective in decreasing HT-29 cell viability. The possible reason for this phenomenon is that C-ori’s amino acid composition (VTFVLIAAK) was rather specific to the HT-29 cell line. Changes or any substitution may have resulted in the failure to inhibit the cancer. Similarly, CTP-ori and CTP-rds were not effective in inhibiting the HT-29 cell line (supplementary Figures S1 and S2). Considering the prediction score of the CTP group and the C group, the SVM scores of the C group were well ahead those of the CTP group. Despite having two modes of action, the CTP group peptides were not the strongest candidates among all peptides from CM, which may have made the peptides unable to successfully inhibit the cancers. However, the explanations behind these incidents will have to be further studied.

Doxorubicin is known to induce apoptosis in cancer cells [26,27]. The apoptosis characteristics in the HT-29 cell line are chromosome condensation (CC), apoptotic bodies (AB), cell membrane blebbing (BL), early apoptosis (EA), and late apoptosis (LA) [28,29]. The effects of C-ori on the HT-29 cell line were similar to those of the doxorubicin according to the AO/PI double staining assay, and C-ori was confirmed to induce apoptosis in the HT-29 cell line. Theoretically, the cationic properties of C-ori could establish non-specific interactions with the negatively charged phospholipids (e.g., phosphatidylglycerol) on cancer cells, which would cause an increase in cell permeability and destabilization of the membrane integrity [30,31,32]. As C-ori was predicted not to be able to disrupt cells intracellularly, its amino acid composition and physiochemical properties may have been the key to the destruction of cells through extracellular actions [33,34]. Furthermore, the decrease in the cholesterol in the cancer cells may have facilitated the apoptosis-induced ACPs to interact with the cells [35,36,37].

Even though there are countless modes of action ACPs can implement to inhibit cancer, some ACPs employ a similar model of the cell-disrupting process to those of antimicrobial peptides (AMPs) [38]. For example, magainin 2 is a bioactive peptide found in the skin secretions of amphibians, that is selective to solid tumors (e.g., the bladder cancer cell line), and utilizes a “carpet model” to destabilize the cells [39,40]. A “carpet-like model” could be the possible mode of action used by C-ori, as this model describes the interaction between positively charged peptides and anionic phospholipids on cancer cells, which means no penetration is involved. C-ori could remain parallel to the cell surface until it reached the critical concentration, and the barrier of the cells was thereafter weakened by the presence of C-ori [41]. Ultimately, the decrease in membrane permeability allowed C-ori to trigger apoptosis and the proliferation of HT-29 was inhibited [42]. However, further research is needed to further evaluate the apoptosis pathway of C-ori on specific molecular pathways, and also with other cancer cell types, e.g., metastasis colorectal cancer or other specific cancer cell lines. Moreover, the non-effective biomimetic ACPs candidates in our work might still have other potential uses as anti-cancer cells, e.g., cell cycle arrest or senescence induction, which are other modes of action that need further different types of cell and molecular analysis.

## 4. Materials and Methods

**Figure 7:**
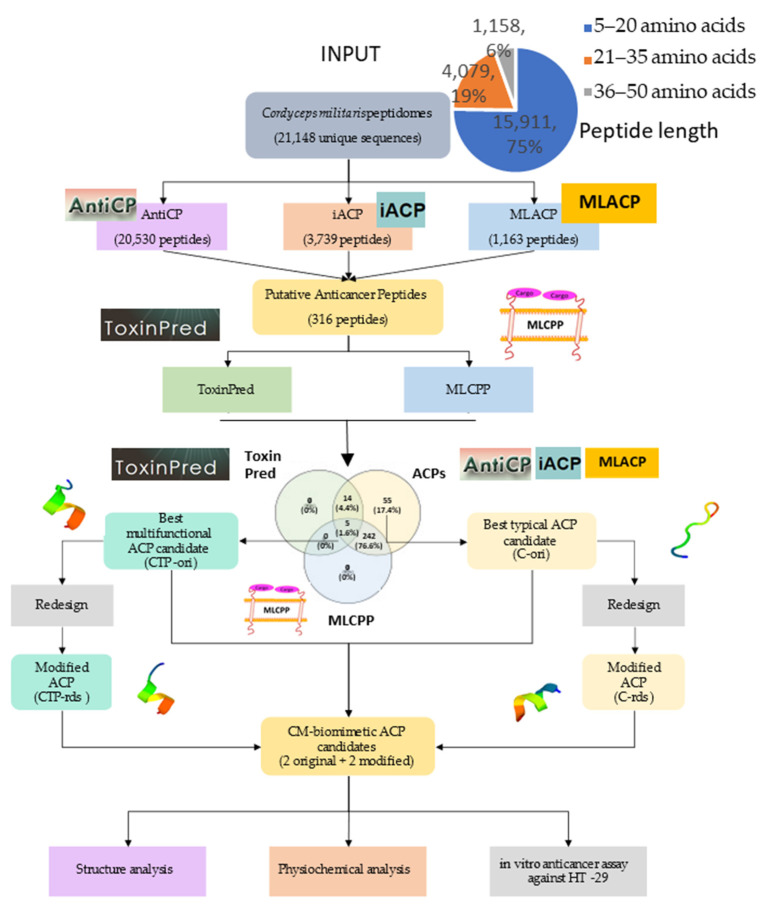
The workflow of the bioinformatic virtual screening for *Cordyceps militaris* (CM) anticancer peptide candidates, and the in vitro analysis of the colon cancer inhibition assay.

As can be seen in the pipeline illustrated in Figure 7, we proposed the bioinformatic virtual screening workflow with an in vitro validation start from stating the inputs of 21,148 putative unique peptide sequences until we selected the top predicted scores of unique ACP groups and multifunctional ACPs (with cytotoxicity and cell-penetrating abilities). Redesigns were also performed to improve the predicted scores, and the biomimetic peptides were experimentally tested with both the non-metastasis colorectal cancer cell line HT-29 and the human dermal fibroblast cell line to ensure the specific effects on cancer cell lines.

### 4.1. The Bioinformatic Prediction of CM Peptides

A total of 21,148 CM peptide sequences were obtained from the predicted cut site of the contig1 CM proteome (from the National Center for Biotechnology Information: NCBI), and the in silico pepsin digestion was performed by our Python scripts. There were 3 groups of peptide length distributions, i.e., 5–20 amino acid residues (15,911 sequences, 75%), 21–35 amino acid residues (4,079 sequences, 19%), and 36–50 amino acid residues (1,158 sequences, 6%) (Figure 7). The peptide sequences were arranged in the FASTA format and were used as the input to predict anticancer properties using 3 machine-learning-based prediction servers, i.e., AntiCP (http://crdd.osdd.net/raghava/anticp, accessed on 12 May 2021), iACP (http://lin-group.cn/server, accessed on 12 May 2021), and MLACP (http://www.thegleelab.org/MLACP/MLACP.html, accessed on 12 May 2021). Venny 2.1.0. (https://bioinfogp.cnb.csic.es/tools/venny, accessed on 12 May 2021) was used to generate the Venn diagram to visualize the unique, dual, and multifunctional peptide candidates from each prediction online bioinformatic program. ToxinPred (https://webs.iiitd.edu.in/raghava/toxinpred/protein.php, accessed on 12 May 2021) and MLCPP (http://www.thegleelab.org/MLCPP, accessed on 12 May 2021) were used to predict whether the peptides were cytotoxic, or if they were cell-membrane-penetrating anti-cancer peptides or not. The selected putative ACPs were redesigned using AntiCP, utilizing the peptide design function. Lastly, PEP-FOLD3.0 (https://bioserv.rpbs.univ-paris-diderot.fr/services/PEP-FOLD3, accessed on 15 May 2021) was used to simulate the feasible molecular structure of the peptides.

### 4.2. Cell Culture

The non-metastasis colorectal cancer cell line HT-29 and the human dermal fibroblast cell were kindly provided as a gift from Dr. Mattaka Khongkaw, senior researcher from National Nanotechnology Center (Nanotec) Thailand. HT-29 and the human dermal fibroblast cell were cultured and passaged in Dulbecco’s Modified Eagle Medium (DMEM, GIBCO—Life Technologies, New York, NY, USA), and supplemented with a 10% heat-inactivated Fetal Bovine Serum (FBS, GIBCO—Life Technologies, NY) at 37 °C under 5% CO_2_.

### 4.3. Determination of Cell Viability by MTT Assay

The cell viability was evaluated by a conventional 3-(4,5-dimethylthiazol-2-yl)-2,5-diphenyltetrazolium bromide (MTT) assay. The selected CM-biomimetic peptides were synthesized by Cellmano Biotech Limited (Hefei, China). The peptides were dissolved by dimethyl sulfoxide (DMSO, Sigma Aldrich, New York, NY, USA) until the final concentration was 100 mM. Cells were preincubated in 96-well plates at 2 × 10^5^ cells/well (100 µL/well) for 24 h, and treated with indicated concentrations of C-ori and C-rds (0 to 400 µM) for 24 h, 48 h, and 72 h, while the cells were treated with CTP-ori and CTP-rds at a different range of concentrations (0 to 100 µM) due to the limited quantity of the peptides. After it was due, MTT was added to each well with the same concentration of 1 mg/mL (10 µL/well) and was further incubated for 4 h. The media were removed and 100 µL of DMSO was added to dissolve the purple crystal. Absorbance was measured at 570 nm using the absorbance microplate reader, PowerWave HT (BioTek Instruments, Berlin, Germany). The cell viability was calculated using the following formula:

(1) $$\%\;\mathrm{cell}\;\mathrm{viability}=\frac{\mathrm{corrected}\;\mathrm{absorbance}\;\mathrm{of}\;\mathrm{treated}\;\mathrm{cells}}{\mathrm{corrected}\;\mathrm{absorbance}\;\mathrm{of}\;\mathrm{control}\;\mathrm{cells}}\times 100$$

The half-maximal inhibitory concentration (IC_50_) was estimated by fitting the data with the non-linear regression equation.

### 4.4. Statistical Analysis of the MTT Assay

Each experiment was conducted at least three times. The data were presented as means ± SD (standard deviation) if not stated otherwise. The parameters were analyzed with a one-way analysis of variance (one-way ANOVA) followed by Dunnett’s multiple comparisons test. Graphpad Prism Version 9.0 (Graphpad Software, California, CA, USA) was utilized to conduct all statistical analysis. The values of *p* < 0.05 and *p* < 0.01 indicated that the differences were statistically significant or highly significant, respectively, when compared to the control group.

### 4.5. Analysis of the Effect of Combination of Peptide and Doxorubicin

The chemotherapy drug, doxorubicin hydrochloride (Tokyo Chemical Industry, Tokyo, Japan), was used to compare the effects with the selected peptide. The condition of the setting experiment was similar to the cell viability determination method. The cells were treated with different concentrations of doxorubicin (0 to 0.25 µM) in a two-fold manner to determine the cytotoxicity of doxorubicin. The combination of C-ori (0 to 400 µM) and doxorubicin (0 to 0.25µM) was treated on cells in a two-fold manner to observe the effect of the combination. The cell viability was evaluated using an MTT assay following the above-mentioned procedures in Section 4.3.

### 4.6. Cell Morphological Study by AO/PI Staining Assay

An acridine orange/propidium iodine (AO/PI) double staining assay was employed to determine the effects of the peptides on the HT-29 cancer cell death. HT-29 was seeded in an 8-well plate at 5 × 10^4^ cells/well (400µL/well), and was allowed to adhere at 37 °C in a 95% relative humidified incubator containing 5% CO_2_ for 24 h. The HT-29 was exposed to different concentrations of various drugs (control; DMSO; doxorubicin 0.25µM; doxorubicin 0.125 µM; C-ori 400 µM, C-ori 200 µM; doxorubicin 0.25 µM + C-ori 400 µM; and doxorubicin 0.125 µM + C-ori 200 µM) for 72 h. The adherent HT-29 cells were trypsinised and centrifuged (Hettich Universal 32 R centrifuge, DJB Labcare Ltd.,London, UK) at 1000 rpm at 4 °C for 10 minutes. The pallet was washed using phosphate-buffered saline (PBS), and re-centrifuged three times with 1000 µL of PBS; afterwards, the PBS and waste were removed. Then, 5 µL of PBS and 5 µL of both 1 mg/mL acridine orange and 10 mg/mL propidium iodine were mixed with the pallet. The aliquots were visualized under a fluorescence microscope (Olympus, Berlin, Germany).

## 5. Conclusions

In conclusion, C-ori was the original pepsinized anticancer peptide from *Cordyceps militaris* which exhibited the apoptogenic effect against non-metastasis colorectal cell lines (HT-29). C-ori can be modified to increase the efficacy of peptides and can be developed to be used as a co-treatment with the conventional chemotherapy in clinical use. Even though the observed anticancer activity of the C-ori was rather weak and not quite suitable for further clinical trials, the anticancer activity of the designed peptide could be improved with drug delivery systems to be more specific to cancer cells with an improved uptake rate. Moreover, the non-effective biomimetic ACP candidates in our work might still have other potential uses as anti-cancer cells, e.g., cell cycle arrest or senescence induction, which are other modes of action that need further investigation. Furthermore, the mechanisms of C-ori in inhibiting other types of various cancer cell lines, as well as the in vivo method conducted on sample animals, have to be further studied.

## Figures and Tables

**Table 1:** Anticancer prediction scores from 3 machine-learning-based prediction programs: AntiCP; iACP; and MLACP. The red highlighted letters indicate the amino acid substitutions.

Peptide			SVM Score	RF Probability	RF-ACP	SVM-ACP
Group	Name	Sequence				
The toxic and cancer cell-penetrating anticancer peptide	CTP-ori	TTMICLTCAR	1.05	0.997	0.515	0.766
	CTP-rds	TTGICLTCCR	1.58	0.997	0.538	0.674
The anticancer peptide	C-ori	VTFVLIAAK	1.28	0.875	0.558	0.787
	C-rds	FTFVLLAAK	1.62	0.943	0.506	0.541

## Data Availability

Not applicable.

## Supplementary Materials

The following are available online, Figure S1: The effect of (a) CTP-ori and (b) CTP-rds on HT-29 cell viability assessed by MTT Assay, Figure S2: The effect of (a) CTP-ori and (b) CTP-rds on fibroblast cell viability assessed by MTT Assay.
