# Supplementary material for: Virtual Screening for Biomimetic Anti-Cancer Peptides from Cordyceps militaris Putative Pepsinized Peptidome and Validation on Colon Cancer Cell Line
Source: Molecules. 2021 Sep 23;26(19):5767. doi: 10.3390/molecules26195767 (PMC8510206; doi:10.3390/molecules26195767)
Supplement: Supplementary file 1 [file molecules-26-05767-s001.zip › molecules-1305805-supplementary.pdf]

molecules

MDPI

Supplementary Material

Virtual Screening for Biomimetic Anti-Cancer Peptides from *Cordyceps militaris* Putative Pepsinized Peptidome and Validation on Colon Cancer Cell Line

Jarinyagon Chantawannakul [1], Paninnuch Chatpattanasiri [1], Vichugorn Wattayagorn [2], Mesayamas Kongsema [2], Tipanart Noikaew [3,*] and Pramote Chumnanpuen [2,4,*]

[1] Mahidol Wittayanusorn School, 364 Salaya, Phuttamonthon District, Nakhon Prathom 73170, Thailand; kiwi.jarinyagon@gmail.com (J.C.); paninnuch.1@gmail.com (P.C.)
[2] Department of Zoology, Faculty of Science, Kasetsart University, Bangkok 10900, Thailand; vichugorn.wa@ku.th (V.W.); fscimmk@ku.ac.th (M.K.)
[3] Department of Biology and Health Science, Mahidol Wittayanusorn School, 364 Salaya, Phuttamonthon District, Nakhon Prathom 73170, Thailand
[4] Omics Center for Agriculture, Bioresources, Food and Health, Kasetsart University (OmiKU), Bangkok 10900, Thailand
* Correspondence: tipanart.noi@mwit.ac.th (T.N.); pramote.c@ku.th (P.C.)

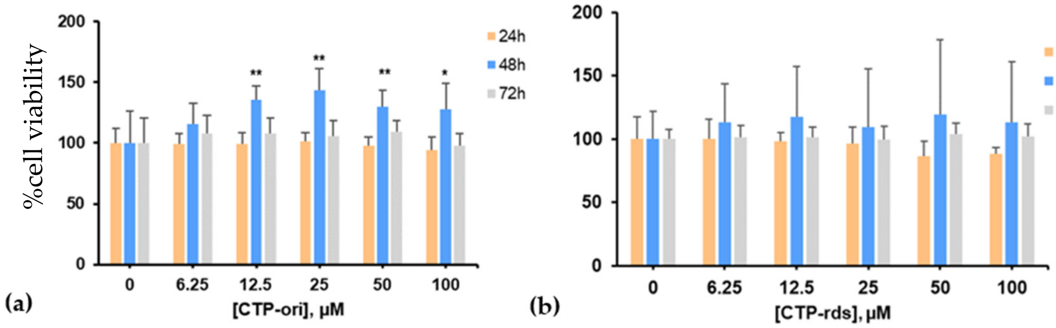

Figure S1. The effect of (**a**) CTP-ori and (**b**) CTP-rds against HT-29 cell viability assessed by MTT Assay. (*$p < 0.05$, ** $p < 0.01$).

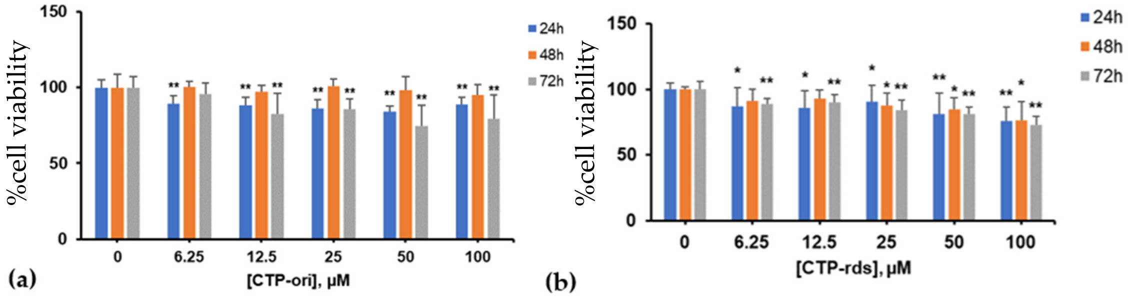

Figure S2. The effect of (**a**) CTP-ori and (**b**) CTP-rds against fibroblast cell viability assessed by MTT Assay. (* $p < 0.05$, ** $p < 0.01$).
